# Investigation on Mass Sensitivity of N-M Type Electrode Quartz Crystal Microbalance

**DOI:** 10.3390/s19092125

**Published:** 2019-05-08

**Authors:** Qiao Chen, Xianhe Huang, Wei Pan, Yuan Xu, Zhichao Fan

**Affiliations:** School of Automation Engineering, University of Electronic Science and Technology of China, Chengdu 611731, China; qiaochen@std.uestc.edu.cn (Q.C.); weipan@std.uestc.edu.cn (W.P.); xuyuan@std.uestc.edu.cn (Y.X.); fanzhichao@std.uestc.edu.cn (Z.F.)

**Keywords:** quartz crystal microbalance (QCM), n-m type electrode, mass sensitivity

## Abstract

Mass sensitivity plays a crucial role in the practical application of quartz crystal microbalances (QCMs)-based quantitative analysis. n-m type QCMs have many applications, so it is necessary to clarify the relationship between the mass sensitivity and the electrode of the n-m type QCM. The performance of gold-plated films with different electrodes was studied by theoretical calculation and experiment. The results show that the mass sensitivity on the surface of the n electrode and the surface of the m electrode are essentially the same. Meanwhile, the mass sensitivity of n-m type QCMs varies with the diameter of the n and m electrodes. When the diameter of the n electrode is close to half the diameter of the m electrode, mass sensitivity is at maximum value. These results are important for the further designs and applications of n-m type QCMs.

## 1. Introduction

Quartz crystal microbalances (QCMs) have gained increasing popularity in various fields, such as chemistry [1,2,3,4,5], biomedicine [6,7,8,9,10], and environmental fields [11,12,13,14], due to high sensitivity, simple operation principles, a digital output, and low-cost components.

**Figure 1:**
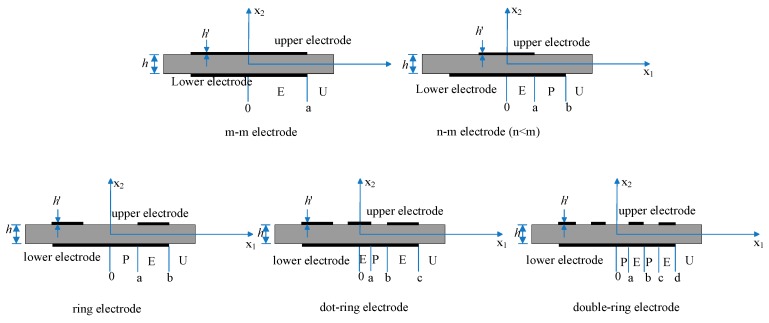
Quartz crystal microbalances (QCMs) with different electrode shapes.

QCMs consist of a disk of the piezoelectric AT-cut quartz sandwiched between two metal excitation electrodes. The QCMs of different electrode shapes are shown in Figure 1. Among various QCMs, n-m type QCMs are commonly used because their quality factor (Q) is higher, which means there are more stable oscillations at particular resonance frequencies, i.e., lower noise levels [15]. The electrode with a small diameter (upper electrode) is denoted as the n electrode, and the electrode with a big diameter (lower electrode) is denoted as the m electrode. 

Mass sensitivity describes the mass-frequency relationship of the surface of the QCM [16] and plays a pivotal role in the quantitative analysis of QCM applications. The mass sensitivity of QCMs with different electrode structures, such as m-m type, n-m type, ring type, dot-ring type, and double ring type electrodes, has been previously studied [11,15,17,18,19,20,21,22]. 

By measuring the frequency change associated with localized mass deposits created by electrochemical deposition, Hillier et al. constructed experimental fitted curves for the mass sensitivity distribution of n-m type QCMs [18]. Through theoretical analysis and experimentation, Josse et al. analyzed the radial dependence of mass sensitivity distribution with different loading factors for n-m type QCMs [15]. Considering that spatially uneven electrodeposition can cause errors between theoretical and experimentally measured mass loads on electrochemical quartz crystal microbalances (EQCM), Kelly et al. discussed the spatial variation of the mass sensitivity factor of n-m type QCMs by changes in the spatial, deposited-mass distribution [21]. To obtain a uniform mass sensitivity distribution, Richardson et al. compared the sensitivity of n-m type QCMs and ring QCMs and constructed a fitted curve for the mass sensitivity of a 5 MHz n-m type QCM [20]. Yang et al. studied the mass sensitivity of the quartz crystal microbalance for various configurations and various thicknesses of the electrodes [19]. 

However, to our knowledge, there are few studies on whether the mass sensitivity of n-m type QCMs on the n electrode and the m electrode are the same. The relationship between the diameter ratio of the n electrode and the m electrode and the mass sensitivity of n-m type QCMs is unclear. These problems have brought difficulties to QCM application analysis. In this article, these issues will be investigated.

## 2. Theory

The practical mass sensitivity has a Gaussian distribution [18,23]. It depends on the particle displacement amplitude function *A*(r,θ) of the QCM and the distance from a given point to the center [17]. The mass sensitivity function of the QCM is defined as follows [11,18,24]:

(1) $$S_{f}\left(r,\theta \right)=\frac{{\left|A\left(r,\theta \right)\right|}^{2}}{2\pi \int_{0}^{\infty }r{\left|A\left(r,\theta \right)\right|}^{2}dr}\cdot C_{f},$$

where $S_{f}\left(r,\theta \right)$ is a mass sensitivity function with a unit of (Hz·kg^−1^), $C_{f}$ is Sauerbrey’s mass sensitivity constant, A(r,θ) is the particle displacement amplitude function and r is the distance from that point to the center. In QCM sensors, the particle displacement amplitude function is independent to the angular direction [15,17]. 

The particle displacement amplitude function A(r) in Equation (1) is a solution of the following Bessel equation [11,15,17]

(2) $$r^{2}\frac{\partial^{2}A}{\partial r^{2}}+r\frac{\partial A}{\partial r}+\frac{k_{i}^{2}r^{2}}{N}A=0,$$

where N depends on material constants of the quartz crystal and $k_{i}^{2}=\left(\omega^{2}-\omega_{i}^{2}\right)/c^{2}$, where i = E, P and U (E, P, and U represent the fully electroded region, partially electroded region, and non-electroded region, respectively); c is the velocity at which waves travel through the crystal. The relationship between c and the material parameters is: $c=\sqrt{\mu_{q}/\rho_{q}}$. $\rho_{q}$ and $\mu_{q}$ are the density and shear modulus of the piezoelectric quartz crystal, respectively; $\omega_{i}$ is cut-off frequency of the fully electroded region ($\omega_{E}$), partially electroded region ($\omega_{P}$), and non-electroded region ($\omega_{U}$), respectively.

The results of the numerical theoretical calculations show that the mass sensitivity of the n electrode and the m electrode are essentially the same. Additionally, consistent with the original results, a decrease in the diameter of the n electrode or the diameter of the m electrode will improve mass sensitivity [25]. However, if the diameter of the n electrode diameter or the diameter of the m electrode is too small, the dynamic resistance (R) will increase; the Q value and frequency stability will be low; at the same time, the particle amplitude will reduce, and mass sensitivity will decrease. Considering the effect of the ratio of the diameters of the two electrodes on mass sensitivity, the results show that the mass sensitivity of n-m type QCMs was at maximum value when the diameter of the n electrode is close to half the diameter of the m electrode.

When the fundamental resonant frequency was 10 MHz, the thickness of the gold electrode with a diameter of 2.5 and 5.1 mm on both sides was 500 Å. By calculation, the mass sensitivity of the AT-cut n-m type QCM is 3.13 × 10^12^ Hz·kg^−1^ at the center of the electrode.

## 3. Experiment

It is difficult to directly detect the mass sensitivity of QCMs. The data on the mass sensitivity of QCMs in the published literature are mostly relative or normalized results [15,26]. Our previous work [27] proposed the equivalent mass sensitivity, which takes into account the Gaussian distribution characteristics and effects of the electrodes on mass sensitivity. Then, a thin film was added onto the surface of the QCM to indirectly verify mass sensitivity distribution. Additionally, the theoretical frequency shift can be calculated according to the following equation:

(3) $$\Delta f=-C_{QCM}^{*}\times \Delta m\;\left(\mathrm{where}\;C_{QCM}^{*}=\frac{1}{\pi r_{d}^{2}}\int_{0}^{r_{d}}2\pi rS_{f}\left(r\right)dr\right),$$

where *r_d_* is the radius of the specified circular region onto which mass load is attached. $C_{QCM}^{*}$ is the equivalent mass sensitivity.

A series of QCM experiments, which plated a rigid gold film onto the surface of the QCM, were performed to investigate the mass sensitivity of n-m type QCMs indirectly. The experimental environment was selected in a class 10,000 ultra-clean room of Wintron Electronic Co., Ltd. (Zhengzhou, China). The ambient temperature in the ultra-clean room was maintained at 23 °C. A total of 36 AT-cut, plano–plano,10-MHz quartz wafers with a diameter of 8.7 mm were used in this experiment. 

**Figure 2:**
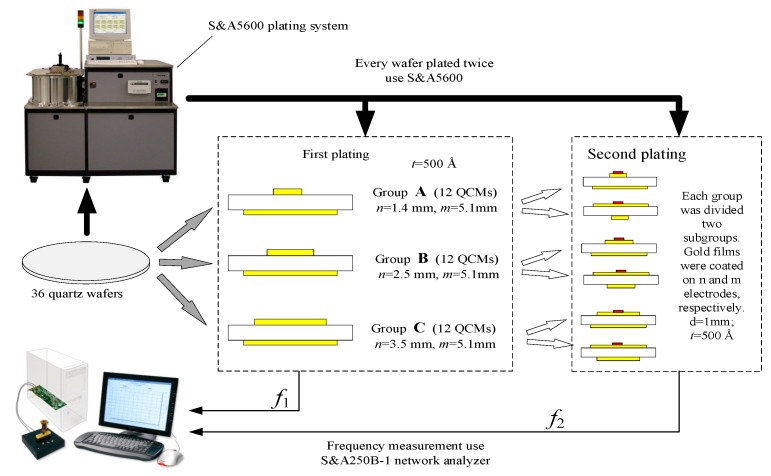
Schematic diagram of the experimental setup.

Figure 2 is a schematic diagram of the experimental set-up. To investigate the mass sensitivity, QCMs were divided into 3 groups according to the different diameters of electrodes. 

In the first plating process, group A was plated gold n electrodes and m electrodes with a diameter of 1.4 and 5.1 mm (QCM-1.4/5.1); group B was plated gold n electrodes and m electrodes with a diameter of 2.5 and 5.1 mm (QCM-2.5/5.1); group C was plated gold n electrodes and m electrodes with a diameter of 3.5 and 5.1 mm (QCM-3.5/5.1). The thickness of all the gold electrodes was 500 Å and their resonant frequencies were measured and recorded as *f*_1_. 

In the second plating process, 12 QCMs for each group, A, B, and C, were divided into two subgroups. A total of 6 QCMs had a coated gold film on the n electrodes with a diameter of 1 mm and a thickness of 500 Å, and the other QCMs were coated on the m electrodes with the same film. Their resonant frequencies were measured and recorded as $f_{2}$. $\Delta f=f_{1}-f_{2}$ was the frequency shift caused by the thin gold film plated in the second plating process. 

The equipment used in the plating process was the S&AW-5600 BASE PLATING SYSTEM (Saunders & Associates, LLC. Phoenix, AZ, USA). The coating thickness was set by the equipment program.

## 4. Results and Discussion

The frequencies of all 36 QCMs (three groups) were measured using the S&A250B-1 network analyzer (Saunders & Associates, LLC. Phoenix, AZ, USA), and the results are shown in Table 1. $\Delta f_{a}$ and $\delta_{a}$ are the average value and the standard deviation of frequency shift, respectively, in each subgroup. $\Delta f_{c}$ and δ are the average value and the standard deviation of frequency shift, respectively, in each group. As is shown in Table 1, all the standard deviations, δ, $\delta_{a}$, were very small, indicating the high stability of the experimental system and the environment.

### 4.1. Effect of Different Electrodes on N-M type QCMs

By comparing frequency changes caused by the second coating process on the n and m electrodes, the results showed that the mass sensitivity of the n electrode surface and m electrode surface were essentially the same. This result is consistent with theoretical calculations, and the error (2% to 4%) between the mass sensitivity of the n electrode surface and m electrode surface may be caused by experimental errors or model errors.

### 4.2. Effect of Different Ratios of Diameters of Electrodes on N-M Type QCMs 

Experimental results also showed that the mass sensitivity of QCM-1.4/5.1 was close to QCM-2.5/5.1. To further analyze the mass sensitivity of QCM-1.4/5.1 and QCM-2.5/5.1, the dynamic resistance R of the equivalent circuit parameters was measured by the S&A250B-1 network analyzer (shown in Table 2).

Δm (about 760 ng) is the mass change caused by the second plating process. Experimental results showed that the mass sensitivity of QCM-1.4/5.1, QCM-2.5/5.1 and QCM-3.5/5.1 is 3.40, 3.45, and 2.99 Hz.ng-1, respectively. By comparing the mass sensitivity of the three groups, the results showed that the mass sensitivity of n-m type QCMs was at maximum value when the diameter of the n electrode was close to half the diameter of the m electrode, which was consistent with the calculation.

Although the mass sensitivity of the QCMs increased as the diameter of the electrode decreased, as R increased, the particle amplitude decreased, so the mass sensitivity decreased. Therefore, under the joint effect of the diameter of the electrode and dynamic resistance, the mass sensitivity of the QCM-1.4/5.1 was lower than QCM-2.5/5.1.

The dynamic capacitance of group B and group C was approximately 7.0 fF and 15.0 fF, and the corresponding quality factor Q was approximately $9.8\times 10^{4}$ and $8.1\times 10^{4}$, respectively. In other words, group B possessed the highest mass sensitivity and a higher Q. So, group B had more advantages than group A and group C.

### 4.3. The Mass Sensitivity of QCM-2.5/5.1 

We continued to analyze the data of group B (QCM-2.5/5.1). According to Equation (3), the equivalent mass sensitivity $C_{QCM}^{*}$ was $2.96\times 10^{12}$ Hz·kg^−1^ at the electrode center within a diameter of 1 mm. So, the theoretical frequency shift $\Delta f_{s}$, caused by Δm (about 760 ng), was 2245 Hz. The relative error $E_{s}$ between $\Delta f_{s}$ and $\Delta f_{c}$ is 14.81%. $E_{s}$ is caused by ignoring the fringing field effect in the theoretical calculation.

## 5. Conclusions

In this paper, through theoretical calculations and experimental verification, the results show that the mass sensitivity of the n electrode surface and the m electrode surface were essentially the same in n-m type QCMs. Meanwhile, the mass sensitivity of n-m type QCMs varied with the diameter of the n and m electrodes. When the diameter of the n electrode was close to half the diameter of the m electrode, the mass sensitivity was at maximum value. This study will provide a reference for further designs and applications of n-m type QCMs.

## Figures and Tables

**Table 1:** Experimental values.

Group	A		B		C	
Electrode	n-Electrode	m-Electrode	n-Electrode	m-Electrode	n-Electrode	m-Electrode
Δf (Hz)	2690	2710	2550	2730	2240	2310
	2590	2670	2600	2680	2240	2330
	2380	2640	2700	2760	2110	2290
	2570	2520	2520	2670	2240	2330
	2630	2540	2400	2640	2320	2300
	2480	2610	2640	2620	2270	2270
$\Delta f_{a}\;\left(\mathrm{Hz}\right)$	2556.67	2615.00	2568.33	2683.33	2236.67	2305.00
$\delta_{a}$ (Hz)	110.94	73.96	104.39	53.17	69.47422	23.45
$\Delta f_{c}$ (Hz)	2585.83		2625.83 ^※^		2270.83	
δ (Hz)	94.91		99.22		60.97	

^※^ The analysis of experimental and theoretical results is shown in Section 4.3.

**Table 2:** Dynamic resistance (R) of all quartz crystal microbalance (QCMs).

Groups	R (Ω)											
A	81.64	77.61	67.19	71.47	67.30	76.77	77.59	68.04	92.04	77.17	97.22	80.04
B	21.42	23.45	21.98	23.98	19.59	19.44	25.37	28.27	24.73	24.46	23.45	22.02
C	12.95	11.35	10.04	12.76	24.61	13.14	11.37	11.33	12.76	11.20	13.02	11.75
